# ﻿ *Gnathiapipinde* sp. nov. (Crustacea, Isopoda, Gnathiidae), a temporary parasite of the pufferfish, *Amblyrhynchoteshonckenii*, from temperate southern Africa

**DOI:** 10.3897/zookeys.1129.90986

**Published:** 2022-11-10

**Authors:** Nico J. Smit, Kerry A. Hadfield

**Affiliations:** 1 Water Research Group, Unit for Environmental Sciences and Management, North-West University, Private Bag X6001, Potchefstroom, 2520, South Africa North-West University Potchefstroom South Africa

**Keywords:** Chintsa, De Hoop Nature Reserve, Indian Ocean, marine fish parasite, taxonomy

## Abstract

A new species, *Gnathiapipinde***sp. nov.**, is described from specimens taken from pufferfish, *Amblyrhynchoteshonckenii*, at Chintsa and De Hoop Nature Reserve on the southern Indian Ocean coast of South Africa. *Gnathiapipinde***sp. nov.** is characterised by the straight frontal margin, presence of conical superior frontolateral process, a strong and bifid mediofrontal processes, pronounced and pointed supraocular lobes, mandible strongly curved with a dentate blade, and the claviform penes produced more than a third the length of the pereon. A summary and key to the males of all known species of the Gnathiidae from the Temperate Southern African marine realm is provided.

## ﻿Introduction

The Western Indian Ocean is a region of known high but underexplored marine invertebrate diversity. The Temperate Southern African (TSA) marine realm (a realm identified by Spalding et al. 2007) is well known for its high biodiversity and endemism of marine fauna, especially fish (Griffiths et al. 2010). However, there is a large gap in our knowledge of the diversity of small invertebrates, including parasites, from all the potential host species already documented (Smit and Hadfield 2015). This is specifically true for gnathiid isopods that are temporary ectoparasites of both teleost and elasmobranch fishes (Hadfield et al. 2008), and only eight species in three genera have previously been reported or described from the TSA (Table 1).

**Table 1.** Summary of the location, depth, size and references of 9 gnathiid species from the Temperate Southern African (TSA) realm, including the 8 previously known species and the new species, *Gnathiapipinde* sp. nov.

Species	Location	Size (mm)	Depth (m)	Substratum / Host	References
*Afrignathiamulticavea* Hadfield & Smit, 2008	South Africa (Eastern and Western Cape)	1.5–2.0	26–73		Hadfield and Smit (2008)
*Caecognathiacryptopais* (Barnard, 1925)	South Africa (Eastern and Western Cape)	2.0–3.9	160		Barnard (1925); Smit et al. (2000)
*Gnathiaafricana* Barnard, 1914	South Africa (Eastern and Western Cape)	0–5.1		Tubes of serpulid worms; sponges; *Caffrogobiuscaffer* (Günther, 1874); *Chorisochismusdentex* (Pallas 1769); *Clinuscottoides* Valenciennes, 1836; *Clinussuperciliosus* (Linnaeus, 1758)	Barnard (1914); Smit and Davies (1999); Smit et al. (1999)
*Gnathiadisjuncta* Barnard, 1920	South Africa (Western Cape)	3.5	73		Barnard (1920)
*Gnathiankulu* Smit & Van As, 2000	South Africa (Eastern Cape)	3.3–4.9	80–200		Smit and Van As (2000)
	Madagascar (Nosy-Be)	4	1.5		Kensley et al. (2009)
*Gnathiapantherina* Smit & Basson, 2002	South Africa (Eastern and Western Cape)	3.7–6.8	intertidal	*Haploblepharusedwardsii* (Schinz, 1822); *Porodermapantherinum* (Müller & Henle, 1838); *Torpedofuscomaculata* Peters, 1855	Smit and Basson (2002)
*Gnathiapilosus* Hadfield, Smit & Avenant-Oldewage, 2008	South Africa (Kwazulu-Natal)	1.6–2.0	intertidal	*Abudefdufsordidus* (Forsskål, 1775); *Acanthurustriostegus* (Linnaeus, 1758); *Antennablenniusbifilum* (Günther, 1861); *Diploduscapensis* (Smith, 1844); *Epinephelusmarginatus* (Lowe, 1834); *Halichoeresnebulosus* (Valenciennes, 1839); *Istiblenniusdussumieri* (Valenciennes, 1836); *Istiblenniusedentulus* (Forster & Schneider, 1801); *Omobranchusbanditus* Smith, 1959; *Plectroglyphidodonleucozonus* (Bleeker, 1859); *Psammogobiusknysnaensis* Smith, 1935; *Pteroismiles* (Bennett, 1828); *Rhabdosargussarba* (Forsskål, 1775); *Scartellaemarginata* (Günther, 1861); *Teraponjarbua* (Forsskål, 1775); *Thalassomapurpureum* (Forsskål, 1775)	Hadfield et al. (2008, 2009)
*Gnathiapipinde* sp. nov.	South Africa (Eastern and Western Cape)	4.6–5.1	Intertidal-shallow; subtidal	*Amblyrhynchoteshonckenii* (Bloch, 1795)	Current study
*Gnathiaspongicola* Barnard, 1920	South Africa (Western Cape)	5	238–347	hexactinellid sponges	Barnard (1920)

These small crustaceans (<15 mm, most species <6 mm) have three larval stages that feed on the blood and lymph of their hosts, before dropping to the ocean floor where they moult into the subsequent larval stage or into adult males or females (Smit et al. 2019). In general, gnathiid larvae are not considered host specific, although too little is known about specific larval species to be conclusive on this point. Due to the highly characteristic structures seen in male gnathiids, the taxonomic classification has been, and still is, based predominantly on the morphology of the adults (Smit and Davies 2004).

Gnathiid isopods have traditionally been described from the free-living adult males collected from dredge sampling, elutriation and sorting of coral rubble, from sponge collections, and other standard techniques used to collect small marine crustaceans. However, since the early 2000s, new gnathiid species have been described by moulting final (P3) larval stages into adults, either collected by light (Farquharson et al. 2012) and baited traps (Shodipo et al. 2021) or collected directly from the host (Ferreira et al. 2010). This latter method also provides the opportunity to obtain morphological and ecological information on the larval stage as well as host information that is generally lacking for most species of gnathiids. Following this trend, this study reports on gnathiid larvae collected from the evileye blaasop (pufferfish), *Amblyrhynchoteshonckenii* (Bloch, 1795), from the south coast of South Africa, and moulted through to adult males. The male gnathiids did not conform to any of the currently known species from this or any other region in the world and are here described as new to science. In addition, a key to the currently known species from this region is provided.

## ﻿Methods

Nine *Amblyrhynchoteshonckenii* were collected from the De Hoop Nature Reserve (4 specimens) and the small coastal town of Chintsa (5 specimens) on South Africa’s south coast during October 1999 and October 2020, respectively. Fish from De Hoop Nature Reserve (34°28'44"S, 20°30'38"E) were captured by luring them into deep pools during evening low tide and then catching them with hand nets (see Smit and Davies 2001). At Chintsa (32°50'01"S, 28°06'58"E), pufferfish were collected using rod and line during early morning low tide. Host nomenclature follows FishBase (Froese and Pauly 2022).

Following capture, live pufferfish were screened for external parasites and fish with gnathiid larvae present were kept alive in fresh, aerated seawater until the gnathiids completed feeding and detached from the host. All the larvae were attached to the dorsal and lateral areas of the body just posterior to the fish’s head. Fish were kept alive until the fully fed larvae detached 6–8 h following capture. In order to rear the larvae into the next stage, they were kept alive in fresh seawater. Free swimming gnathiids were collected by pipettes and transferred into small 50 ml bottles with fresh seawater until moulting into adults (see Grutter et al. 2020). Three of the larvae moulted 9 d post-feeding. A subsample of larvae and adults were subsequently fixed in 75% ethanol and transferred to the laboratory for further analysis.

From the collected samples, a single third stage larva and male were cleaned and prepared for scanning electron microscopy (SEM). The fixed specimens were hydrated from 70% ethanol to fresh water. The organisms were then washed and cleaned by brushing them with a soft sable hairbrush to remove salt crystals and debris. Clean specimens were dehydrated through a series of ethanol concentrations and critical-point dried using standard techniques. Dried specimens were mounted on inverted conical stubs with a rapid-drying varnish (Japan Gold Size, Winsor and Newton) normally used in gilding. Specimens were sputter coated with gold and studied with the aid of a JEOL WINSEM JSM 6400 scanning electron microscope. Optimum results were obtained when SEM work was done at 10 kV with a working distance of 39 mm and the stage tilted at 70° to 90°. For light microscopy, temporary slides of lignin pink stained specimens were prepared as whole mounts or dissected appendages. These were examined with a Leitz Laborlux D compound and a Wild M5 dissection microscope and drawings were made from projections using drawing attachments on these microscopes.

The species descriptions of both male and larva were prepared in DELTA (DEscriptive Language for TAxonomy) using a modified Gnathiidae character set (as used in Hadfield et al. 2019) for the male and a newly generated character set for the larva. Terminology follows Monod (1926), Cohen and Poore (1994), and Svavarsson and Bruce (2012, 2019) for the male and Smit and Basson (2002) and Hadfield et al. (2008) for the larva. Isopod classification follows that of Brandt and Poore (2003), and setal terminology is based on Watling (1989) and Svavarsson and Bruce (2019). The habitus total length was measured mid-dorsally, from the frontal margin (excluding the mandibles) to the midpoint of the pleotelson. Appendages (mouthparts, pereopods, pleopods) were dissected from the same side of the isopod.

Research permits for fish collection were provided by the Department of Agriculture, Forestry and Fisheries (DAFF) (RES2019/103 and RES2020/29), and ethical clearance was through the North-West University AnimCare animal ethics committee (NWU-00440-16-A5 and NWU-0051-19-A5). Type material is deposited in the National Museum, Bloemfontein (NMB), South Africa.

## ﻿Taxonomy

**Suborder Cymothoida Wägele, 1989**

**Superfamily Cymothooidea Leach, 1814**

### ﻿Family Gnathiidae Leach, 1814

#### ﻿ Gnathia

Taxon classificationAnimaliaIsopodaGnathiidae

Leach, 1814

D9197297-9887-5F8E-BCA5-A4025EF14457

##### Type species.

*Gnathiatermitoides* Leach, 1814, by monotypy (Cohen and Poore 1994).

##### Remarks.

A restricted synonymy and diagnosis of *Gnathia* was provided in Hadfield et al. (2019).

#### ﻿ Gnathia pipinde sp. nov.

Taxon classificationAnimaliaIsopodaGnathiidae

7817FDC5-0FCA-56A9-9423-24A4BEE4C6DE

https://zoobank.org/1D3309F5-2048-4E33-A8A4-308C7B523852

**Figure 1.**
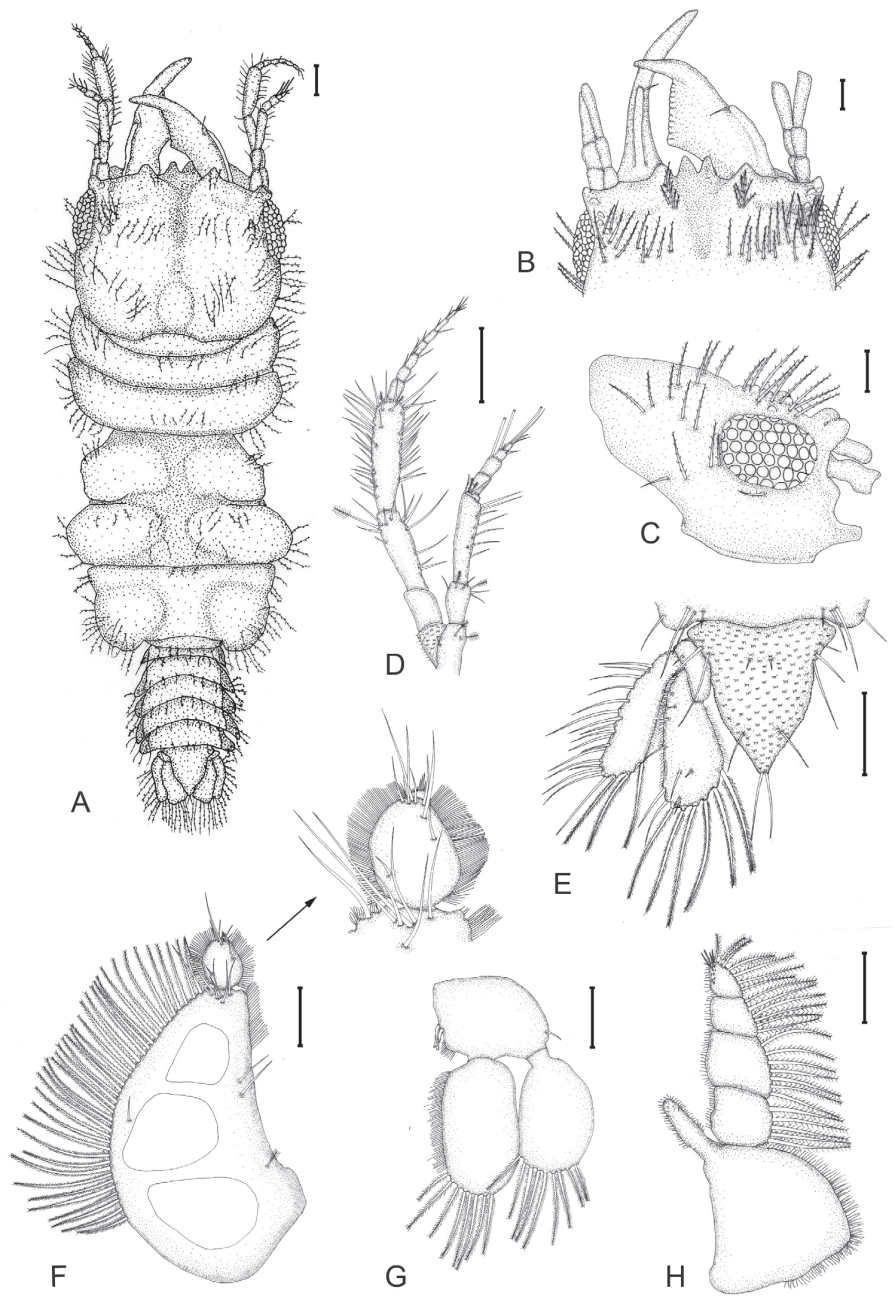
*Gnathiapipinde* sp. nov. (NMB P 899), male holotype (4.6 mm TL) **A** habitus dorsal view **B** dorsal view of frontal border and mandibles **C** lateral view of cephalosome **D** antenna and antennula **E** pleotelson and uropod **F** pylopod **G** pleopod 2 **H** maxilliped. Scale bars: 200 μm.

**Figure 2.**
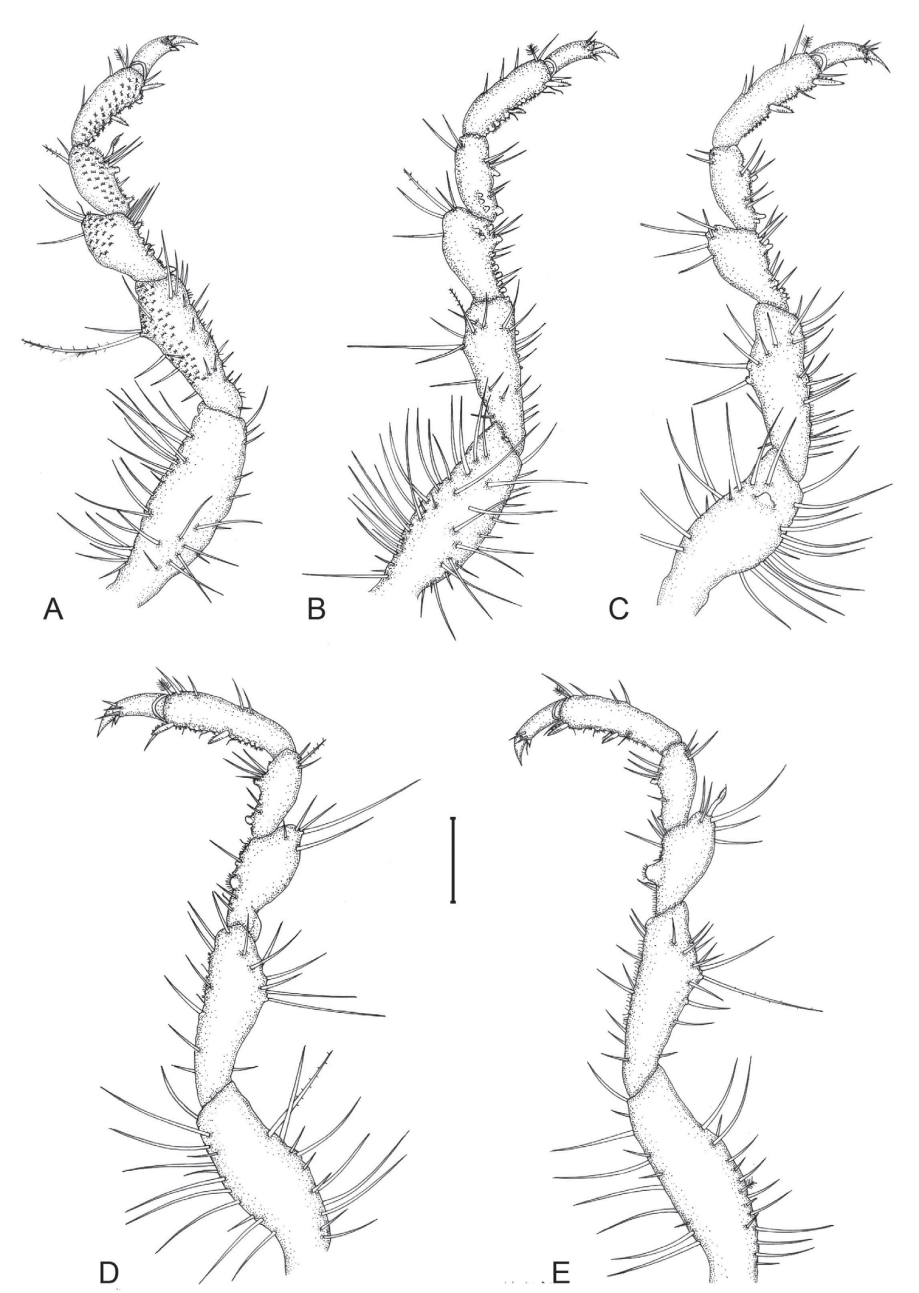
*Gnathiapipinde* sp. nov. (NMB P 899), male holotype (4.6 mm TL) **A–E** pereopods 2–6, respectively. Scale bar: 200 μm.

**Figure 3.**
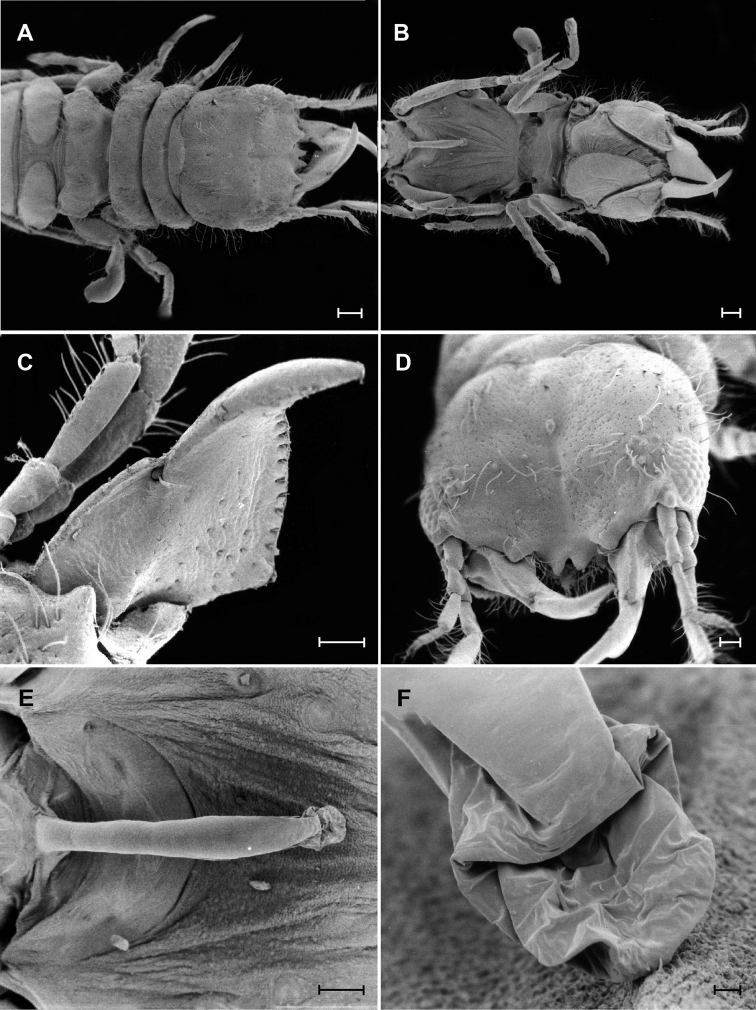
*Gnathiapipinde* sp. nov. (NMB P 900), male scanning electron microscope images. **A** Dorsal view of cephalosome and pereon **B** ventral view of cephalosome and pereon **C** dorsal view of mandible **D** anterodorsal view of cephalosome **E** ventral view of penes **F** ventral view of anterior end of penes. Scale bars: 250 μm (**A**, **B**); 100 μm (**C**–**E**); 10 μm (**F**).

**Figure 4.**
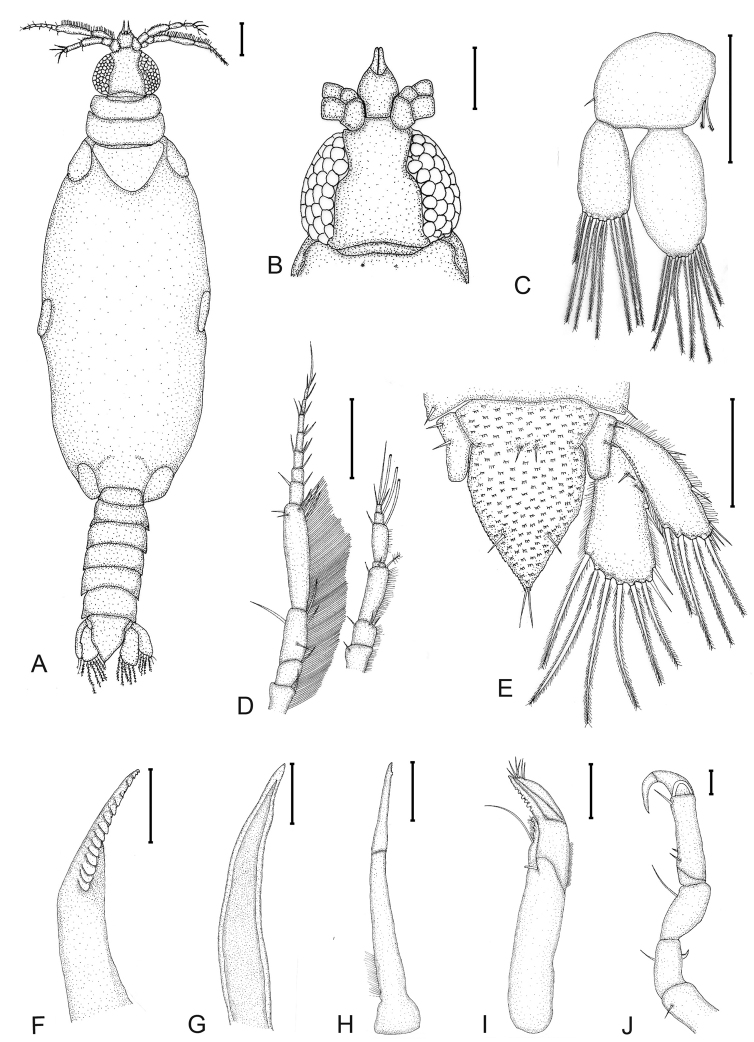
*Gnathiapipinde* sp. nov. (NMB P 901), praniza larva (4.1 mm TL) **A** habitus dorsal view **B** dorsal cephalosome **C** pleopod 1 **D** antenna and antennula **E** pleotelson and uropod **F** mandible **G** paragnath **H** maxillula **I** maxilliped **J** gnathopod. Scale bars: 200 μm (**A–E**); 50 μm (**F–J**).

**Figure 5.**
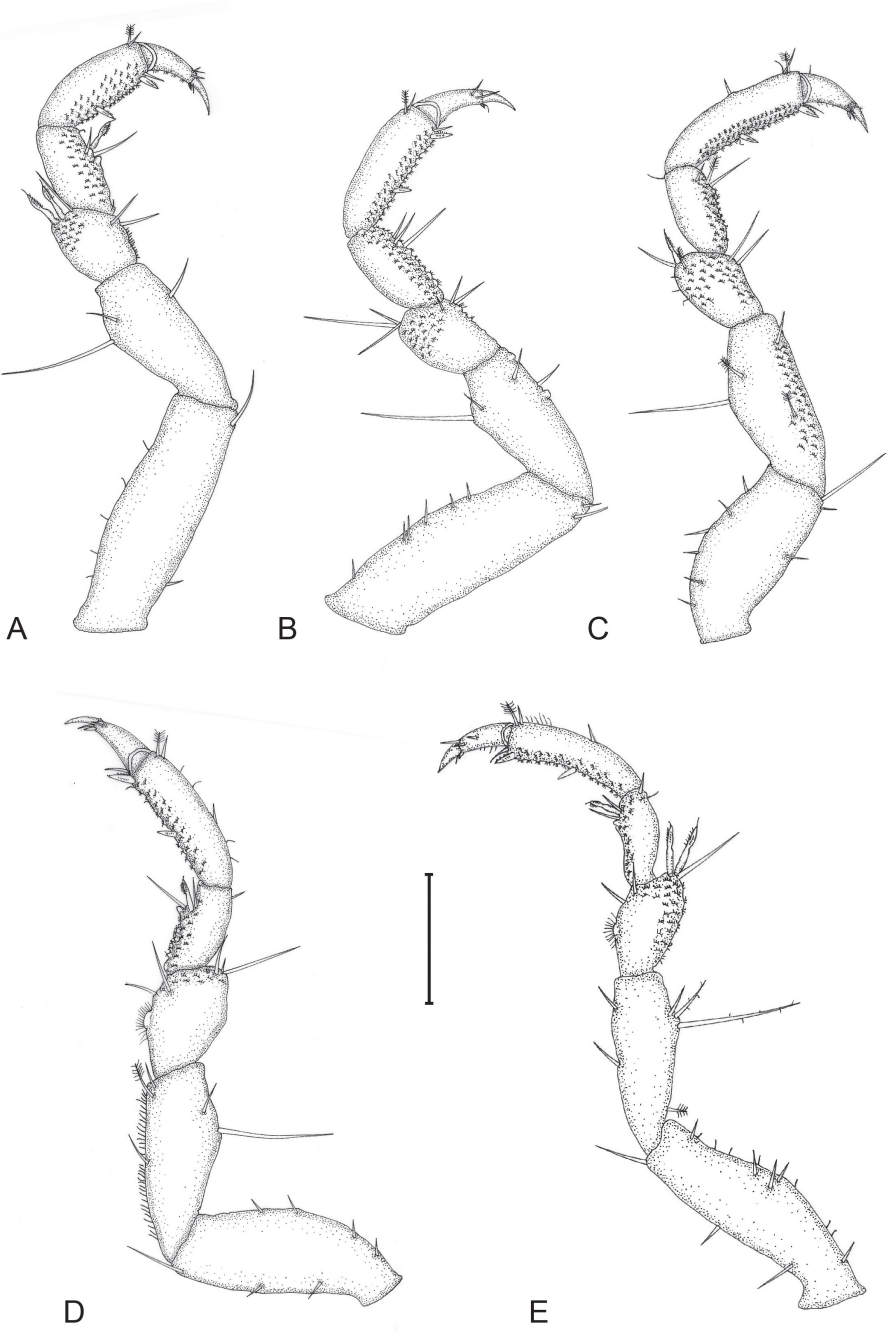
*Gnathiapipinde* sp. nov. (NMB P 901), praniza larva (4.1 mm TL) **A–E** pereopods 2–6, respectively. Scale bar: 200 μm.

**Figure 6.**
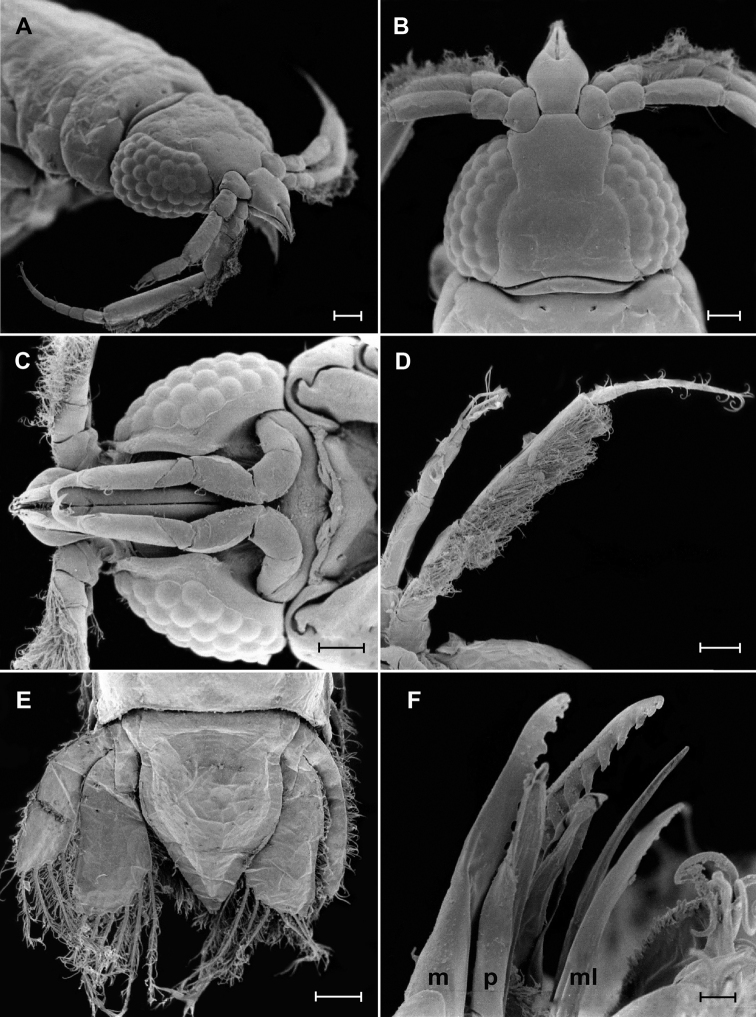
*Gnathiapipinde* sp. nov. (NMB P 901), praniza larva scanning electron microscope images **A** anterolateral view of labrum, cephalosome, and antennae **B** dorsal view of cephalosome **C** ventral view of cephalosome **D** ventral view of antenna and antennula **E** dorsal view of the pleotelson and uropods **F** lateral view of mandible (m), paragnath (p), and maxillula (ml). Scale bars: 100 μm (**A–E**); 10 μm (**F**).

Figs 1 , 2 , 3 , 4 , 5 , 6

##### Material examined.

***Holotype***. South Africa • 1 ♂ (4.6 mm TL, dissected); De Hoop Nature Reserve (34°28'44"S, 20°30'38"E); October 1999; leg. N.J. Smit; from *Amblyrhynchoteshonckenii* (NMB P 899). ***Paratypes***. South Africa • 1 ♂ used for SEM (5.1 mm TL), 11 praniza larvae (P3) (3.8–4.6 mm TL), 1 P3 larva for SEM (4.2 mm TL); same info as holotype (NMB P 900) • 2 praniza larvae (P3) (3.8, 4.3 mm TL); Chintsa (32°50'01"S, 28°06'58"E); October 2020; leg. K.A. Hadfield; from *Amblyrhynchoteshonckenii* (NMB P 901).

##### Description of male adult.

(Figs 1–4). *Body* 2.8 times as long as greatest width, widest at pereonite 5; dorsal surfaces punctate, sparsely setose. *Cephalosome* quadrate, 0.8 as long as wide, lateral margins subparallel, posterior margin concave; dorsal surface with sensory pits and sparse granules; dorsal sulcus narrow, deep, short; paraocular ornamentation weakly developed, posteromedian tubercle present. *processes* present. *Frontal margin* straight, median point with processes. *External scissura* present, narrow, shallow. *Mediofrontal process* present, strong, bifid, without fine setae. *Superior frontolateral process* present, single, strong, conical, with 6 pairs of long simple setae. *Inferior frontolateral process* absent. *Supraocular lobe* pronounced, pointed; accessory supraocular lobe not pronounced. *Eyes* present, elongate, 0.4 as long as cephalosome length, contiguous with head surface, ommatidia arranged in rows.

*Pereon* lateral margins subparallel, with few setose setae; anteriorly smooth. *Pereonite 1* partially fused dorsally with cephalosome; dorsolateral margins partly obscured by cephalosome. *Pereonite 2* wider than pereonite 1. *Pereonite 4* with prominent anterior constriction separating it from pereonite 3, median groove present. *Areae laterales* present on pereonite 5. *Pereonite 6* with weak lobi laterales; lobuii absent or weak, conical. *Pleon* epimera dorsally visible on all pleonites. *Pleonite* lateral margins with 2 pairs of simple setae, long setose setae randomly distributed on posterior margins. *Pleotelson* as long as anterior width, covered in pectinate scales; lateral margins finely serrate, anterolateral margins concave, with 3 submarginal setae; posterolateral margin weakly convex, with 2 submarginal setae; mid-dorsal surface with 2 submedian setae, apex with 2 setae.

*Antennula* shorter than antenna; peduncle article 1 with 2 penicillate setae; article 2 0.8 times as long as article 1, with 2 penicillate setae; article 3 2.1 times as long as article 2, 3.8 times as long as wide. Antennula flagellum 0.8 times as long as article 3, with 5 articles; article 1 with 2 plumose setae; article 3 with 1 aesthetasc seta; article 4 with 1 aesthetasc seta, and 2 simple setae; article 5 terminating with 1 aesthetasc seta, and 3 simple setae. *Antenna* peduncle article 1 covered in pectinate scales and marginal setae; article 3 2.9 times as long as wide, twice as long as article 2, with 1 penicillate seta, and 12–14 simple setae and 1 penicillate seta; article 4 1.3 times as long as article 3, 3.7 times as long as wide, with 2 penicillate setae, and with 40–50 simple setae; flagellum as long as article 4, with 7 articles, terminating with 4 simple setae.

*Mandible* 0.6 as long as width of cephalosome, rectangular, strongly curved, distally; apex cylindrical, 22% total length, distally raised in lateral view; mandibular seta present. *Carina* present, smooth. *Incisor* knob-like. *Blade* present, dentate, straight, dentate along 100% of margin, with tufts of setae along distal margin. Basal neck short; sensory pits with short simple hair-like setae distributed randomly on dorsal surface of blade. Pseudoblade, internal lobe and dorsal lobe absent; erisma present; lamina dentata absent.

*Maxilliped* 5-articled, mesial border with short simple hair-like setae; article 1 lateral margin with continuous marginal short setae, endite extending to distal margin of article 2, without coupling setae; article 2 lateral margin with 5 plumose setae; article 3 lateral margin with 7 plumose setae; article 4 lateral margin with 6 plumose setae; article 5 with 7 plumose setae and 5 simple setae.

*Pylopod* article 1 with three distinct areolae, 1.6 as long as wide, without distolateral lobe; posterior and lateral margins forming rounded curve; lateral margin with 38 large plumose setae; mesial margin with scale-setae on distal part only, 2 simple setae and 1 penicillate seta; distal margin with 6 simple setae; article 2 1.3 as long as wide; with 8 simple setae; article 3 minute, with 4 simple setae.

*Pereopods 2–6* with long simple setae and randomly covered in pectinate scales (only illustrated on pereopod 2); inferior margins with weak tubercles. *Pereopod 2* with tubercles on merus and carpus; basis 2.9 times as long as greatest width, superior margin with 19 setae, inferior margin with 12 setae; ischium 0.7 times as long as basis, 2.5 as long as wide, superior margin with 6 simple setae and 1 long setose seta, inferior margin with 17 setae; merus 0.5 as long as ischium, 1.5 as long as wide, superior margin with bulbous protrusion and 4 setae, inferior margin with tubercles and 7 setae; carpus 0.5 as long as ischium, 1.8 as long as wide, superior margin with 2 setae (1 setose), inferior margin with tubercles and 5 setae (1 serrate seta); propodus 0.7 times as long as ischium, 2.5 times as long as wide, superior margin with 3 simple setae and 1 penicillate seta, inferior margin with 2 simple setae and 2 robust setae; dactylus 0.7 as long as propodus, terminates in sharp posterior pointing unguis. *Pereopods 3 and 4* similar to pereopod 2. *Pereopod 5* similar to pereopod 6. *Pereopod 6* with tubercles on merus and carpus; basis 3 times as long as greatest width, superior margin with 14 setae, and 1 penicillate seta, inferior margin with 11 setae; ischium 0.9 as long as basis, 3.2 as long as greatest width, superior margin with 12 setae, inferior margin with 8 setae; merus 0.5 as long as ischium, 1.8 times as long as wide, superior margin with 6 setae, inferior margin with 4 setae, without dense patch of scale-setae; carpus 0.5 as long as ischium, 2.2 times as long as wide, superior margin with 3 setae, inferior margin with 4 setae; propodus 0.6 as long as ischium, 3.8 times as long as wide, superior margin with 5 setae, inferior margin with 5 setae, and 2 robust setae; dactylus 0.6 as long as propodus.

*Penes* produced, more than a third length of pereon; penial processes 6.5 times as long as basal width, anterior end ending in sac-like extension dorsal to opening.

*Pleopod 2 exopod* 1.7 as long as wide, distally broadly rounded, with 9 plumose setae; endopod 1.8 as long as wide, distally broadly rounded, with 8 plumose setae; appendix masculina absent; peduncle 0.8 times as wide as long, mesial margin with 2 coupling setae, lateral margin with 1 simple seta.

*Uropod* rami extending beyond pleotelson, apices narrowly rounded. *Peduncle* with 1 dorsal seta. *Uropod endopod* 2.4 as long as greatest width, dorsally with 4 setae; lateral margin straight, lateral margin with 6 simple setae; proximomesial margin weakly convex, with 6 long plumose setae. *Uropod exopod* not extending to end of endopod, 3.3 times as long as greatest width; lateral margin weakly sinuate, with 17 simple setae; proximomesial margin straight, distally convex, with 3 long plumose setae.

##### Description of praniza 3 larva.

(Figs 4–6). *Body* 3.5 times as long as greatest width; dorsal surfaces sparsely punctate, sparsely setose. *Cephalosome* 0.7 times as long as wide; posterior margin slightly concave; lateral margins convex; dorsal surface with few sensory pits and no setae; anterior margin straight with lateral concave excavations to accommodate first articles of antennae. *Labrum* prominent, 0.4 times as long as cephalosome, semicircular with apical processes, anterior margin concave. *Eyes* well developed, oval-shaped; almost as long as cephalosome; one eye 0.3 as wide as cephalosome, bulbous, standing out from head surface; ommatidia arranged in rows; eye colour black.

*Pereon* elongate, 2.3 times as long as wide, smooth, with no setae or sensory pits. *Pereonite 1* partially fused dorsally with cephalosome; dorsally visible; dorsolateral margins partly obscured by cephalosome. *Pereonite 2 and 3* similar in size and shape. *Pereonite 4* triangular, 2.1 times as wide as long, posterior margin stretching over pereonite 5, lateral shields at leg attachment. *Pereonite 5* consists of elastic membrane fully expanded in praniza stage with blood meal, lateral shields at leg attachment. *Pereonite 6* rectangular, posterior margin slightly concave, lateral shields at leg attachment. *Pereonite 7* dorsally visible; posterior margin rounded, overlapping pleonite 1. *Pleon* with all 5 pleonites dorsally visible; pleon and pleotelson 0.4 times as long as pereon. *Pleonite 5* almost twice the length of the other articles. *Pleotelson* 1.3 times as long as anterior width, covered in pectinate scales; lateral margins finely serrate; anterolateral margins concave, without submarginal setae; posterolateral margin weakly convex, with 2 submarginal setae; mid-dorsal surface with 2 submedian setae, apex with 2 setae.

*Antennula* shorter than antenna; peduncle with short hair-like setae on anterior borders of all three articles; article 2 with 3 setae, as long as article 1; article 3 with 2 setae (1 penicillate), 2.3 times as long as article 2, 2.9 times as long as wide. Antennula flagellum 1.1 times as long as article 3, with 4 articles; article 1 with 2–3 setae; article 2 with 1 aesthetasc seta and 1 simple seta, 0.6 times as long as flagellum; article 3 with 1 aesthetasc seta; article 4 terminating with 1 aesthetasc seta and 3 simple setae. *Antenna* with rows of long simple setae on anterior margins of all 4 peduncle articles; peduncle article 3 2.3 times as long as wide, 2 times as long as article 2, with 4 simple setae; article 4 twice as long as article 3, 4.2 times as long as wide, with 6 simple setae; flagellum 1.1 times as long as article 4, with 7 articles, terminating with 3 or 4 simple setae.

*Mandible* stout, distal margin styliform with 16 teeth on mesial margin (3 smaller at tip of mandible), conical and posteriorly directed, increasing in size from anterior to posterior.

*Paragnaths* elongate, gutter-like, terminates in sharp point, no teeth.

*Maxillula* long, slender, 6–8 small teeth on distal inner margin, lateral border with marginal setae proximally.

*Maxilliped* cylindrical with elongated base, endite extending to distal margin of palp article 2, with 1 long simple seta and coupling seta. *Maxilliped palp* 3-articled; article 1 with 6 teeth mesially, lateral margin with setae; article 3 with 5–7 simple setae.

*Gnathopod* smaller than pereopods, with 7 articles, carpus reduced, few simple setae on articles 1–5, 1 robust seta on article 2.

*Pereopods 2–6* with long simple setae, pectinate scales covering inner margins of propodus and carpus, and outer margins of merus. *Pereopod 2* with tubercles on carpus; basis 2.6 times as long as greatest width, superior margin with 5 setae, inferior margin with 2 setae; ischium 0.7 times as long as basis, twice as long as wide, superior margin with 2 setae, inferior margin with 1 seta; merus 0.5 as long as ischium, 1.3 as long as wide, superior margin with bulbous protrusion, with 3 setae (2 serrate) and pectinate scales, inferior margin with 2 setae; carpus 0.5 as long as ischium, 1.6 as long as wide, superior margin with no setae, inferior margin with 4 setae (1 serrate) and pectinate scales; propodus 0.7 times as long as ischium, 2.3 times as long as wide, superior margin with 2 setae (1 penicillate), inferior margin with 1 simple seta, 2 robust setae and pectinate scales; dactylus 0.9 as long as propodus, with 5 setae, terminates in sharp posterior pointing unguis. *Pereopods 3 and 4* similar to pereopod 2 (differ in setation). *Pereopod 5* similar to pereopod 6. *Pereopod 6* with tubercles on carpus; basis 3.3 times as long as greatest width, superior margin with 12 setae, inferior margin with 3 setae; ischium 0.7 as long as basis, 2.5 as long as greatest width, superior margin with 4 setae (1 penicillate, 2 setose), inferior margin with 2 setae; merus 0.6 as long as ischium, 1.4 times as long as wide, superior margin with bulbous protrusion and 3 setae (2 serrate), inferior margin with 2 setae, with dense patches of pectinate scales; carpus 0.6 as long as ischium, 2.2 times as long as wide, superior margin with 2 setae, inferior margin with 3 setae (1 serrate) and pectinate scales; propodus 0.8 as long as ischium, 3.6 times as long as wide, superior margin with 9 setae, inferior margin with 1 seta, 2 robust setae, and pectinate scales; dactylus 0.6 as long as propodus, with 4 setae, terminates in sharp posterior pointing unguis.

*Pleopod 1 exopod* 1.9 as long as wide, distally broadly rounded, with 8 plumose setae; endopod 1.8 as long as wide, distally narrowly rounded, with 7 plumose setae; peduncle 0.8 times as wide as long, mesial margin with 2 coupling setae, lateral margin with 1 simple seta.

*Uropod* rami extending to pleotelson apex, apices narrowly rounded, fringes with short simple setae. *Peduncle* with 2 dorsal setae. *Uropod endopod* 1.9 as long as greatest width, dorsally with 3 setae; lateral margin straight, lateral margin with 3 simple setae; proximomesial margin weakly convex, with 6 long plumose setae. *Uropod exopod* not extending to end of endopod, 3 times as long as greatest width; lateral margin weakly convex, with 5 simple setae; proximomesial margin straight, distally concave, with 4 long plumose setae.

##### Etymology.

The Xhosa word “pipinde” was chosen, because “pipi” means penis and the post “nde” means long in the language of this southern African tribe and thus referring to the most distinct characteristic of this species. Pronounced as pie-pie-n-dê. The species epithet is a noun in apposition.

##### Distribution.

South coast of South Africa (Western and Eastern Cape Provinces).

##### Hosts.

*Amblyrhynchoteshonckenii* (Bloch, 1785).

##### Remarks.

*Gnathiapipinde* sp. nov. can be identified by the straight frontal margin; presence of conical superior frontolateral process; a strong and bifid mediofrontal processes; pronounced and pointed supraocular lobes; mandible strongly curved with a dentate blade; and the claviform penes produced more than a third the length of the pereon.

When compared to the other known South African gnathiid species, the shape and size of the frontal process of *G.pipinde* are very similar to those of *G.africana*Barnard 1914 (see Smit et al. 1999) and *G.nkulu* Smit & Van As, 2002 (see Smit and Van As 2002). This species can, however, easily be distinguished from the other South African congeners by the shape and size of its elongated penes, narrowing at the distal end with a sac-like extension.

There are currently 134 species of *Gnathia* known to science (Boyko et al. 2008 onwards). To the authors’ knowledge there are nine other *Gnathia* species with elongate penes that differ from each other in shape in size, namely: *Gnathiacamuripenis* Tanaka, 2004; *Gnathiacooki* Müller, 1989; *Gnathiadentata* (G.O. Sars, 1872); *Gnathiafalcipenis* Holdich & Harrison, 1980; *Gnathiafallax* Monod, 1926; *Gnathiainopinata* Monod, 1926; *Gnathialimicola* Ota & Tanaka; 2007; *Gnathiaphallonajopsis* Monod, 1925; and *Gnathiasomalia* Kensley, Schotte & Poore, 2009. Of these, only three resemble that of *G.pipinde* sp. nov. These are *G.falcipenis* from the Great Barrier Reef, Australia; *G.cooki* from Morea, Society Islands in the tropical Pacific; and *G.camuripenis* from southern Japan and the Philippines (Shodipo et al. 2021). According to Holdich and Harrison (1980), the mediofrontal processes of *G.falcipenis* is conical and, thus, distinctly different from the bifid mediofrontal processes of *G.pipinde* sp. nov. The superior frontolateral process and shape of the pleotelson of *G.cooki* described by Müller (1989) are very similar to those of *G.pipinde* sp. nov. However, in the South African species there are six long simple setae on each superior frontolateral processes and the inferior frontolateral process are absent, whereas in the species from the Pacific there are only three long simple setae and the inferior frontolateral process are present. In addition, *G.pipinde* sp. nov. differs in the number of antenna flagellum articles (seven instead of six), and the number of plumose setae on the distal four articles of the maxillipeds (in the order of 5-7-6-7 instead of 5-6-5-7 as in *G.cooki*). The males of *G.camuripenis* can easily be distinguished from *G.pipinde* sp. nov. in its rounded mediofrontal processes without a ventral notch and large bifid internal lobe present on the mandibles (Tanaka 2004).

The larvae of *G.pipinde* sp. nov. can be distinguished from the described larvae of the South African species, *G.africana* and *G.pantherina* Smit & Basson, 2002, by the presence of 16 teeth on the mandible (*G.africana* with nine or 10 and *G.pantherina* with eight). The shape of the pleotelson of *G.pipinde* (posterior two-thirds convex) also differs from that of *G.africana* (lateral margins straight) and *G.pantherina* (anterior half of lateral margins slightly concave).

This species is host-specific to *Amblyrhynchoteshonckenii* and only occurs on the temperate south coast of South Africa (with low intensity and rare infections), despite the wide distribution of the host fish (temperate south coast to subtropical east coast of South Africa). The specific locality preference is also observed with another parasitic isopod on this host fish, *Cinusatetrodontis* Schioedte & Meinert, 1884. Hadfield et al. (2010) reported this species from *A.honckenii*, also only from the temperate south coast of South Africa. This specific area seems to be a parasite diversity hotspot within the TSA realm and could still yield other unknown parasites with further research. Information on the nine known gnathiid species from the TSA is summarised in Table 1, along with a key to these species.

### ﻿Key to species of the family Gnathiidae known from the Temperate Southern African (TSA) realm

This key is based on the morphological characters of the adult male:

**Table:** 

1	Cephalosome wider than pereon; frontal border concave	***Gnathiaspongicola* Barnard, 1920**
–	Cephalosome not wider than pereon; frontal border produced	**2**
2	Frontal border rounded, strongly produced, without any frontal process	***Caecognathiacryptopais* (Barnard, 1925)**
–	Frontal border slightly produced, with frontal process	**3**
3	Pylopod single-articled; two rows of teeth on mandible blade	***Afrignathiamulticavea* Hadfield & Smit, 2008**
–	Pylopod 2- or 3-articled; absent or single row of teeth on blade	**4**
4	Mediofrontal process bifid	**5**
–	Mediofrontal process absent or not bifid	**6**
5	Penes produced, more than a third length of pereon	***Gnathiapipinde* sp. nov.**
–	Penes not produced, two contiguous papillae as long as wide	***Gnathiaafricana* Barnard, 1914**
6	Mandibles short and stout (<0.5 length of cephalosome)	***Gnathiapilosus* Hadfield, Smit & Avenant-Oldewage, 2008**
–	Mandibles long (>0.5 length of cephalosome)	**7**
7	Pereonite 5 completely separated into two lateral halves (pereonite 6 in contact with pereonite 4)	***Gnathiadisjuncta* Barnard, 1920**
–	Pereonite 5 not separated (pereonite 6 not in contact with pereonite 4)	**8**
8	Pereonite 5 areae laterales absent, narrower than pereonite 6; pleopod 2 with appendix masculina > 0.5 length of rami	***Gnathiankulu* Smit & Van As, 2000**
–	Pereonite 5 areae laterales present, as wide or wider than pereonite 6; pleopod 2 with appendix masculina < 0.5 length length of rami	***Gnathiapantherina* Smit & Basson, 2002**

## Supplementary Material

XML Treatment for Gnathia

XML Treatment for Gnathia pipinde
